# A Modular Fusion Protein Strategy Confers Broad-Spectrum Resistance Against *Phytophthora infestans*, *Alternaria solani*, and *Ralstonia solanacearum*

**DOI:** 10.3390/jof12090676

**Published:** 2026-09-10

**Authors:** Kun Yang, Rongbo Wang, Liguang Liu, Kang An, Jitao Liu, Li Wang, Jianwei Shan, Chengchen Li, Liang Qi, Li Zheng, Xiaobo Li

**Affiliations:** 1 Guangdong Provincial Key Laboratory of Crop Genetic Improvement, Crops Research Institute, Guangdong Academy of Agricultural Sciences, Guangzhou 510640, China; ankang@gdaas.cn (K.A.); liu-j-t@163.com (J.L.); wangli20090617@163.com (L.W.); chanyujianwei@126.com (J.S.); lccazdb@163.com (C.L.); qiliang@gdaas.cn (L.Q.); 2 Fujian Key Laboratory for Monitoring and Integrated Management of Crop Pests, Institute of Plant Protection, Fujian Academy of Agricultural Sciences, Fuzhou 350013, China; wrb16128@163.com; 3 Key Laboratory of Green Prevention and Control on Fruits and Vegetables in South China, Ministry of Agriculture and Rural Affairs, College of Agriculture and Biology, Zhongkai University of Agriculture and Engineering, Guangzhou 510225, China; llgwx156@163.com (L.L.); bluestar183@163.com (L.Z.)

**Keywords:** broad spectrum, fusion protein, plant resistance

## Abstract

Potato production is severely threatened by the oomycete *Phytophthora infestans*, the fungus *Alternaria solani*, and the bacterium *Ralstonia solanacearum*; however, strategies capable of simultaneously managing these three pathogen classes remain relatively scarce. In this study, we designed a series of signal peptide (SP)-fused recombinant constructs containing four functional modules with distinct antimicrobial and immune-inducing properties: GAFP1-2×FYVE, an oomycete-inhibiting antimicrobial protein; BbAFP1-ErBD, a fungal-suppressive protein fragment; the pathogen-associated molecular pattern (PAMP) csp22 derived from *R. solanacearum*, which elicits effective anti-bacterial immunity; and the microbe-associated molecular pattern (MAMP) PpEli2 identified from *Pythium periplocum*, which triggers broad-spectrum plant defense responses. Through systematic evaluation of six module order rearrangements, we identified SP-cBG as the optimal multi-domain combination. Further optimization via the introduction of a rigid alpha-helical linker (HL4) yielded SP-cBG-HL4, which tended to show superior resistance against all three pathogens, as reflected in smaller lesion diameters and decreased bacterial titers. qRT-PCR analyses revealed that SP-cBG-HL4 significantly upregulated PTI (*CYP71D20*, *PTI5*), SA (*PR1*, *PR2*), and JA/ET (*PR3*, *PR4*) defense marker genes. Collectively, these findings show that modular domain assembly combined with linker-mediated modular combinatorial optimization represents a powerful engineering strategy for achieving broad-spectrum disease resistance, offering a promising approach to simultaneously control oomycete, fungal, and bacterial pathogens of plants.

## 1. Introduction

Potato (*Solanum tuberosum*) is the world’s most important non-cereal food crop, ranking third in global food production after wheat and rice, and serves as a critical pillar for ensuring food security for billions of people worldwide [1,2,3]. However, its production is severely constrained by three major destructive diseases: late blight caused by the oomycete *Phytophthora infestans*, early blight incited by the fungal pathogen *Alternaria solani*, and bacterial wilt induced by the soil-borne bacterium *Ralstonia solanacearum* [4,5,6,7]. *P. infestans*, the notorious pathogen responsible for the mid-19th-century Irish Potato Famine, continues to inflict annual economic losses exceeding $10 billion globally [3,8,9]. Potato early blight, caused by *A. solani*, can lead to yield losses ranging from 5% to 40%, as the severe foliar necrotic lesions directly disrupt leaf function and reduce the accumulation of photosynthetic assimilates [10,11,12]. Meanwhile, *R. solanacearum*, which is capable of causing disease in more than 250 plant species, triggers rapid wilting and plant death following infection in potatoes, with yield reductions exceeding 80% in severe cases [4,13,14]. Currently, the management of these diseases is heavily reliant on the frequent and widespread application of synthetic agrochemicals. Nevertheless, the excessive use of these compounds poses substantial risks to environmental sustainability, human health, and occupational safety. Consequently, there is an urgent need to explore sustainable alternatives and develop innovative disease management strategies to safeguard potato production.

Breeding for disease resistance is a sustainable strategy, yet broad-spectrum resistance remains a major bottleneck, as genetic resources conferring simultaneous resistance against oomycetes, fungi, and bacteria are relatively scarce. To date, most reported strategies have predominantly targeted a single pathogen class. For instance, the antimicrobial protein GAFP1-2×FYVE has been identified as an effective module against oomycete pathogens, as we demonstrated in our previous studies [15]. Similarly, the antifungal peptide BbAFP1-ErBD functions as a potent fungal-suppressive protein fragment [16,17]. On the antibacterial front, the bacterial-derived pathogen-associated molecular pattern (PAMP) csp22, originating from *R. solanacearum*, has been demonstrated to activate robust antibacterial immunity in planta [4]. Beyond these pathogen-specific strategies, some broad-spectrum immune inducers have been characterized. Notably, we previously showed that the microbe-associated molecular pattern (MAMP) PpEli2, derived from *Py. periplocum*, is capable of triggering broad-spectrum plant defense responses [18]. Despite these advances, strategies capable of simultaneously managing oomycete, fungal, and bacterial pathogens remain relatively scarce.

In this study, we designed and evaluated 12 secreted recombinant fusion constructs by combining four functional modules with distinct antimicrobial and immune-eliciting activities, including GAFP1-2×FYVE, BbAFP1-ErBD, csp22, and PpEli2. We systematically investigated the effects of modular arrangement and linker design on resistance against three major phytopathogens—*P. infestans*, *A. solani*, and *R. solanacearum*—in *N. benthamiana*. Furthermore, we examined the expression levels of defense-related marker genes to gain insights into the underlying immune responses. Our objectives were to (i) identify the optimal modular combination and order for broad-spectrum resistance, (ii) determine whether linker optimization could further enhance efficacy, and (iii) evaluate the expression patterns of marker genes associated with PTI, SA, and JA/ET signaling pathways in response to different constructs. The results of this study may provide valuable insights into the rational design of multi-module fusion proteins for engineering broad-spectrum disease resistance in plants.

## 2. Materials and Methods

### 2.1. Plant Growth Conditions and Microbial Strain Cultivation

*N. benthamiana* seeds were sown in soil and grown in a controlled growth chamber at 25 °C under long-day conditions (16 h light/8 h dark). 5- to 8-week-old plants with fully expanded leaves were used for agroinfiltration experiments [19]. *P. infestans* isolate MZ15-30 was maintained on rye agar medium supplemented with 2% (*w*/*v*) sucrose at 18 °C in the dark [20]. *A. solani* isolate NHL-192 was cultured on potato dextrose agar (PDA) medium at 25 °C in the dark [21]. *R. solanacearum* isolate GD-126 was grown on Luria–Bertani (LB) agar medium at 28 °C in the dark [22].

### 2.2. Vector Construction and Fusion Gene Design

**Figure 1:**
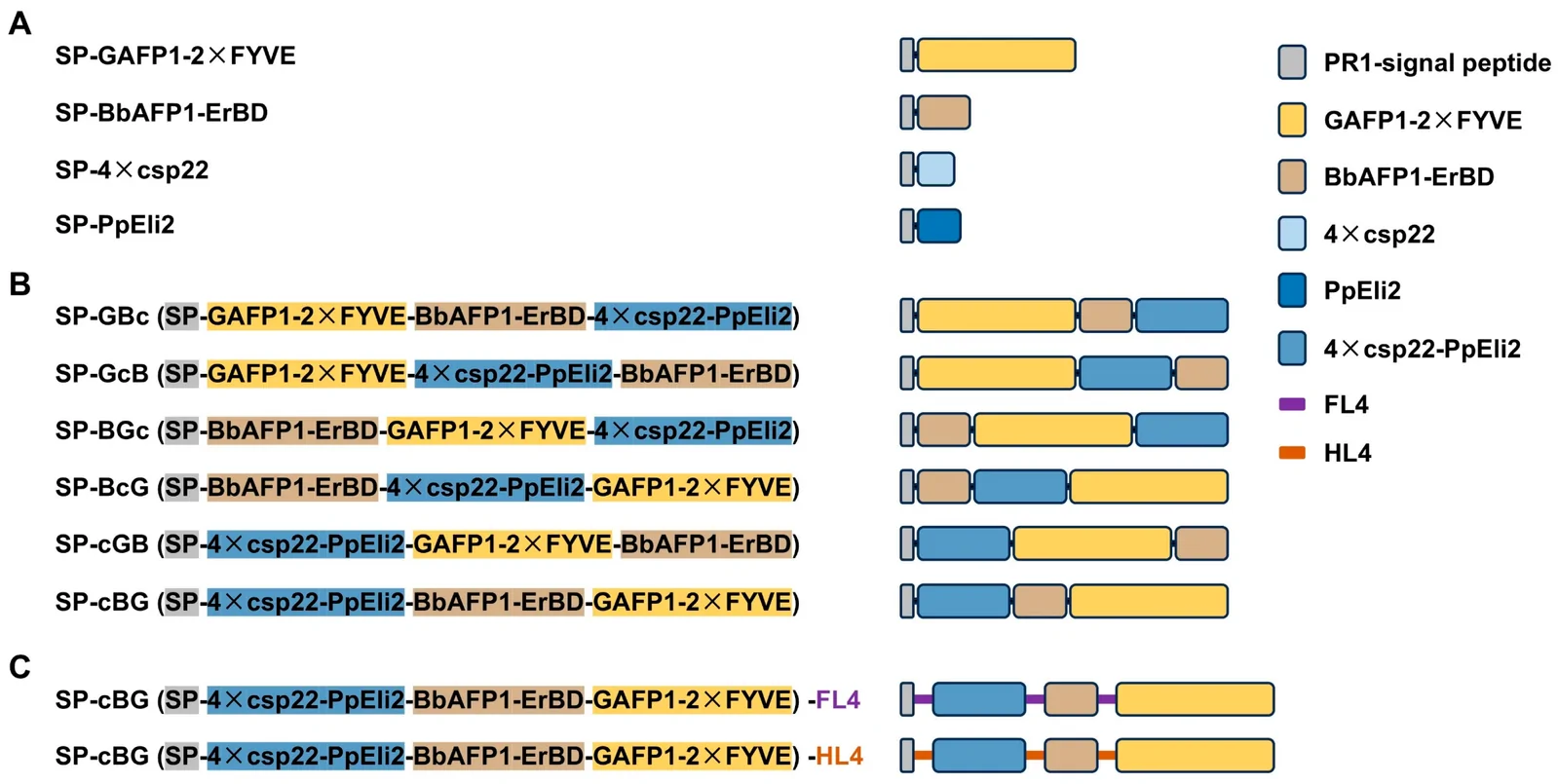
Schematic diagrams of the 12 secreted recombinant construct designs: (**A**) All constructs were fused with the PR1-derived signal peptide (SP) at the N-terminus for extracellular secretion. Individual functional modules are defined as follows: SP-GAFP1-2×FYVE encodes an oomycete-inhibiting antimicrobial protein module; SP-BbAFP1-ErBD functions as a fungal-suppressive protein fragment; SP-4×csp22 is a bacterial-derived PAMP from *Ralstonia solanacearum* that activates antibacterial immunity; and SP-PpEli2 is an MAMP identified from *Pythium periplocum* capable of triggering broad-spectrum plant defense responses. Single-module standalone constructs: SP-GAFP1-2×FYVE, SP-BbAFP1-ErBD, SP-4×csp22, and SP-PpEli2. (**B**) Six multi-module tandem fusion constructs were generated by rearranging the order of these modules. SP-GBc: SP-GAFP1-2×FYVE-BbAFP1-ErBD-4×csp22-PpEli2; SP-GcB: SP-GAFP1-2×FYVE-4×csp22-PpEli2-BbAFP1-ErBD; SP-BGc: SP-BbAFP1-ErBD-GAFP1-2×FYVE-4×csp22-PpEli2; SP-BcG: SP-BbAFP1-ErBD-4×csp22-PpEli2-GAFP1-2×FYVE; SP-cGB: SP-4×csp22-PpEli2-GAFP1-2×FYVE-BbAFP1-ErBD; SP-cBG: SP-4×csp22-PpEli2-BbAFP1-ErBD-GAFP1-2×FYVE. (**C**) Two SP-cBG-derived linker-modified variants: SP-cBG-FL4 fused with flexible linker FL4 and SP-cBG-HL4 fused with rigid linker HL4.

The fusion gene constructs SP-GAFP1-2×FYVE, SP-BbAFP1-ErBD, SP-4×csp22, SP-PpEli2, SP-GBc, SP-GcB, SP-BGc, SP-BcG, SP-cGB, and SP-cBG were synthesized and assembled by Rugao Silicon BioScience Co., Ltd. The constructs SP-cBG-FL4 and SP-cBG-HL4 were synthesized and assembled by RootPath (with offices in Framingham, MA, USA, and Guangzhou, China). All fusion gene sequences were subcloned into the binary vector pBINHA via the SmaI restriction site. Each construct was designed with an N-terminal *PR1* signal peptide for apoplast secretion, followed by one of the four genes (*GAFP1-2×FYVE*, *BbAFP1-ErBD*, *csp22*, or *PpEli2*) or various combinations thereof, arranged in the configurations illustrated in Figure 1. The coding sequences of all genes were based on previously published reports [4,15,16,17,18]. A flexible linker (LGGGGSGGGGSGGGGSGGGGSAAA) or a rigid linker (LAEAAAKEAAAKEAAAKEAAAKAAA) was introduced between functional domains as indicated in Figure 1 [23].

### 2.3. Agrobacterium-Mediated Transient Expression in N. benthamiana

*A. tumefaciens* strain GV3101 was employed for transient expression assays in *N. benthamiana*. Binary constructs were introduced into GV3101 via electroporation. Transformed agrobacteria harboring the target expression vectors were cultured overnight at 28 °C and 200 rpm in Luria–Bertani (LB) liquid medium supplemented with kanamycin (50 mg L^−1^) and rifampicin (50 mg L^−1^). Bacterial cells were pelleted by centrifugation at 4500 rpm for 3 min and then washed three times with infiltration buffer (10 mM MgCl_2_, 10 mM MES, pH 5.7, and 200 µM acetosyringone). Fully expanded leaves of 5- to 8-week-old *N. benthamiana* plants were infiltrated with a 1:1 (*v/v*) mixture of *Agrobacterium* cultures carrying the expression construct and the p19 viral suppressor of RNA silencing, adjusted to an optical density at 600 nm (OD_600_) of 0.4 [19,24].

### 2.4. P. infestans, A. solani, and R. solanacearum Inoculation

For *P. infestans* inoculation, sporangia were harvested from 10-day-old rye agar cultures, resuspended in cold sterile water to 4 × 10^4^ sporangia mL^−1^, and 20 μL droplets of the suspension were inoculated onto *N. benthamiana* leaves. Inoculated leaves were placed under dark, high-humidity conditions for 4 days. Disease severity was assessed by measuring lesion diameter and lesion area for each inoculation site [15,20]. For *A. solani* inoculation, mycelial plugs (5 mm diameter) from 7-day-old PDA cultures were placed upside down onto the center of each leaf disc. Inoculated discs were incubated at 25 °C in the dark for 4 days, and lesion diameter and lesion area were measured [21]. For *R. solanacearum* inoculation, strain GD-126 was cultured overnight in LB medium at 28 °C, harvested, and resuspended in 10 mM MgCl_2_ to 1 × 10^7^ CFU mL^−1^. The suspension was infiltrated into agroinfiltrated *N. benthamiana* leaves using a needleless syringe. At 24 h post-inoculation, leaf discs (0.5 cm diameter) were collected, ground in 100 μL sterile water, serially diluted, and plated on selective LB agar. Colonies were counted after 24–48 h at 28 °C [22]. For inoculation experiments with *P. infestans*, *A. solani*, and *R. solanacearum*, each treatment was applied to 30 independent leaves as technical replicates per experimental run (*n* = 30, technical replicates), and three independent experimental runs were performed as biological replicates (*N* = 3, biological replicates) for each pathogen. All leaves used were the 3rd to 5th leaves from plants at the same developmental stage, ensuring relatively consistent leaf size throughout the experiments, and all inoculation assays were performed at 36 h post-agroinfiltration.

### 2.5. RNA Extraction and Quantitative Real-Time PCR (RT-qPCR) Analysis

Total RNA was extracted from *N benthamiana* leaf tissues (*n* = 3 biological replicates per sample). Frozen leaf materials were ground to a fine powder in liquid nitrogen, and total RNA was subsequently isolated using the E.Z.N.A.^®^ Plant RNA Kit (Omega Bio-Tek, Norcross, GA, USA) according to the manufacturer’s instructions. The RNA concentration and purity were determined using a spectrophotometer (NanoDrop 2000, Thermo Fisher Scientific, Waltham, MA, USA). For cDNA synthesis, 1 µg of total RNA from each sample was reverse-transcribed using the HiScript II Q RT SuperMix for qPCR (+gDNA wiper) (Vazyme Biotech Co., Ltd., Nanjing, China), which includes a genomic DNA elimination step to prevent contamination. The resulting cDNA was diluted 10-fold and stored at −20 °C until further use. RT-qPCR was performed on an ABI 7500 Fast Real-Time PCR system (Applied Biosystems, Foster City, CA, USA) using the ChamQ SYBR Color qPCR Master Mix (Vazyme Biotech Co., Ltd., China). Each 20 µL reaction contained 10 µL of 2× Master Mix, 0.4 µM of each gene-specific primer, and 2 µL of diluted cDNA template. The PCR cycling conditions were as follows: initial denaturation at 95 °C for 30 s, followed by 40 cycles of 95 °C for 5 s and 60 °C for 30 s. A melting curve analysis was performed to confirm the specificity of amplification. The translation elongation factor 1 alpha (*EF-1α*) gene was used as the endogenous reference control to normalize gene expression. The primers used for all target genes and the reference gene are listed in Table 1. Relative gene expression levels were calculated using the 2^−ΔΔCT^ method, with the control sample group serving as the calibrator. All reactions were performed in technical triplicates [19].

### 2.6. Statistical Analysis

Statistical analyses were performed using GraphPad Prism (version 10.4.1). Normality and log-normality of data distributions were evaluated across groups via the Shapiro–Wilk test (α = 0.05). All data passed the Shapiro–Wilk normality test (α = 0.05), confirming that they followed a normal distribution. For datasets conforming to a normal distribution, intergroup comparisons across multiple groups were carried out using one-way analysis of variance (ANOVA), followed by Tukey’s multiple-comparison test for all pairwise comparisons. All statistical analyses, including one-way ANOVA and Tukey’s multiple-comparison tests, were performed using the mean values obtained from three independent experimental runs (*N* = 3) as the experimental units. Technical replicates (*n* = 30 per run) were used solely to calculate the means and standard deviations presented in the figures and were not treated as independent observations for hypothesis testing. Relative resistance increases versus GFP (%) were calculated as (A − B)/A × 100, where A is the lesion diameter or area of the GFP control, and B is the lesion diameter or area of the tested construct.

## 3. Results

### 3.1. Design and Modular Architecture of 12 Secreted Recombinant Fusion Constructs

To enable extracellular secretion, all constructs were fused at the N-terminus with a signal peptide (SP) derived from PR1. Four functional modules with distinct antimicrobial or immune-eliciting activities were employed: SP-GAFP1-2×FYVE, encoding an oomycete-inhibiting antimicrobial protein; SP-BbAFP1-ErBD, a fungal-suppressive protein fragment; SP-4×csp22, a bacterial-derived PAMP from *R. solanacearum* that activates antibacterial immunity; and SP-PpEli2, an MAMP from *Py. periplocum* capable of triggering broad-spectrum plant defense responses. Four single-module standalone constructs were generated (Figure 1A). To explore potential synergistic effects, six multi-module tandem fusion constructs were generated by rearranging the order of these four modules, designated as SP-GBc, SP-GcB, SP-BGc, SP-BcG, SP-cGB, and SP-cBG (Figure 1B). Furthermore, two linker-modified variants based on SP-cBG were constructed by introducing either a flexible linker (FL4) or a rigid linker (HL4), yielding SP-cBG-FL4 and SP-cBG-HL4, respectively (Figure 1C).

### 3.2. Resistance Evaluation of 12 Fusion Constructs Against P. infestans

**Figure 2:**
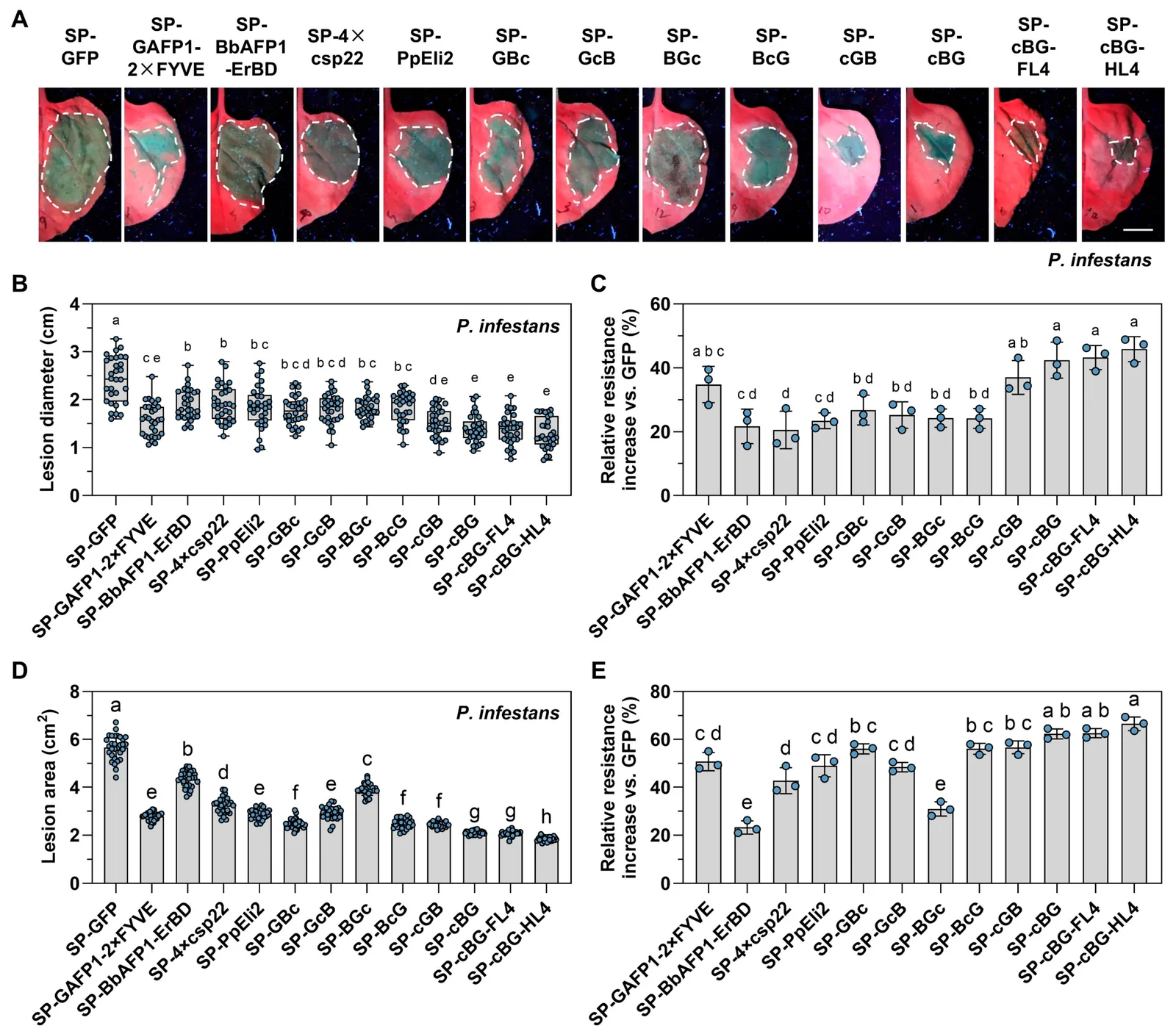
Evaluation of resistance conferred by 12 fusion constructs against *P. infestans*: (**A**) Representative disease lesion phenotypes on *N. benthamiana* leaves expressing the indicated SP-fusion constructs upon *P. infestans* inoculation at 4 days post-inoculation (dpi). Scale bar, 1 cm. (**B**) Quantification of lesion diameter triggered by *P. infestans* on leaves expressing different fusion constructs (*n* = 30 technical replicates). (**C**) Relative resistance increase (%) of each construct compared with the SP-GFP control, calculated based on lesion diameter data in (**B**) (*N* = 3 biological replicates). (**D**) Lesion area measurement of *P. infestans*-infected leaves for the 12 fusion constructs (*n* = 30 technical replicates). (**E**) Relative resistance increase (%) normalized to SP-GFP, derived from lesion area statistics in (**D**) (*N* = 3 biological replicates). Data are presented as mean ± SD. The Shapiro–Wilk test (α = 0.05) was used to assess the normality and log-normality of data distributions across groups. One-way ANOVA was performed for normally distributed datasets, followed by Tukey’s multiple-comparison test for all pairwise comparisons. Different lowercase letters indicate significant differences among treatments (*p* < 0.05). All experiments were repeated three times with consistent results.

To assess the efficacy of these constructs against oomycete pathogens, we transiently expressed each of the 12 SP-fusion constructs in *N. benthamiana* leaves followed by inoculation with *P. infestans*. At 4 dpi, distinct lesion phenotypes were observed among the different constructs (Figure 2A). Quantitative analysis of lesion diameters revealed that all fusion constructs significantly reduced lesion size compared to the SP-GFP control. Among them, SP-cBG, SP-cBG-FL4, and SP-cBG-HL4 exhibited the smallest lesion diameters, indicating the best disease resistance (Figure 2B). Correspondingly, the relative resistance increases calculated from lesion diameter data showed that these three constructs had the highest values (Figure 2C). For lesion area measurements, SP-cBG-HL4 conferred the greatest reduction, while SP-cBG and SP-cBG-FL4 also demonstrated substantial decreases (Figure 2D). Notably, the relative resistance increases derived from lesion area statistics revealed that although SP-cBG-HL4 ranked the highest, its difference from SP-cBG and SP-cBG-FL4 was not statistically significant (Figure 2E). Collectively, these results demonstrate that SP-cBG, SP-cBG-FL4, and SP-cBG-HL4 represent the most effective constructs among the 12 designs.

### 3.3. Resistance Evaluation of 12 Fusion Constructs Against A. solani and R. solanacearum

**Figure 3:**
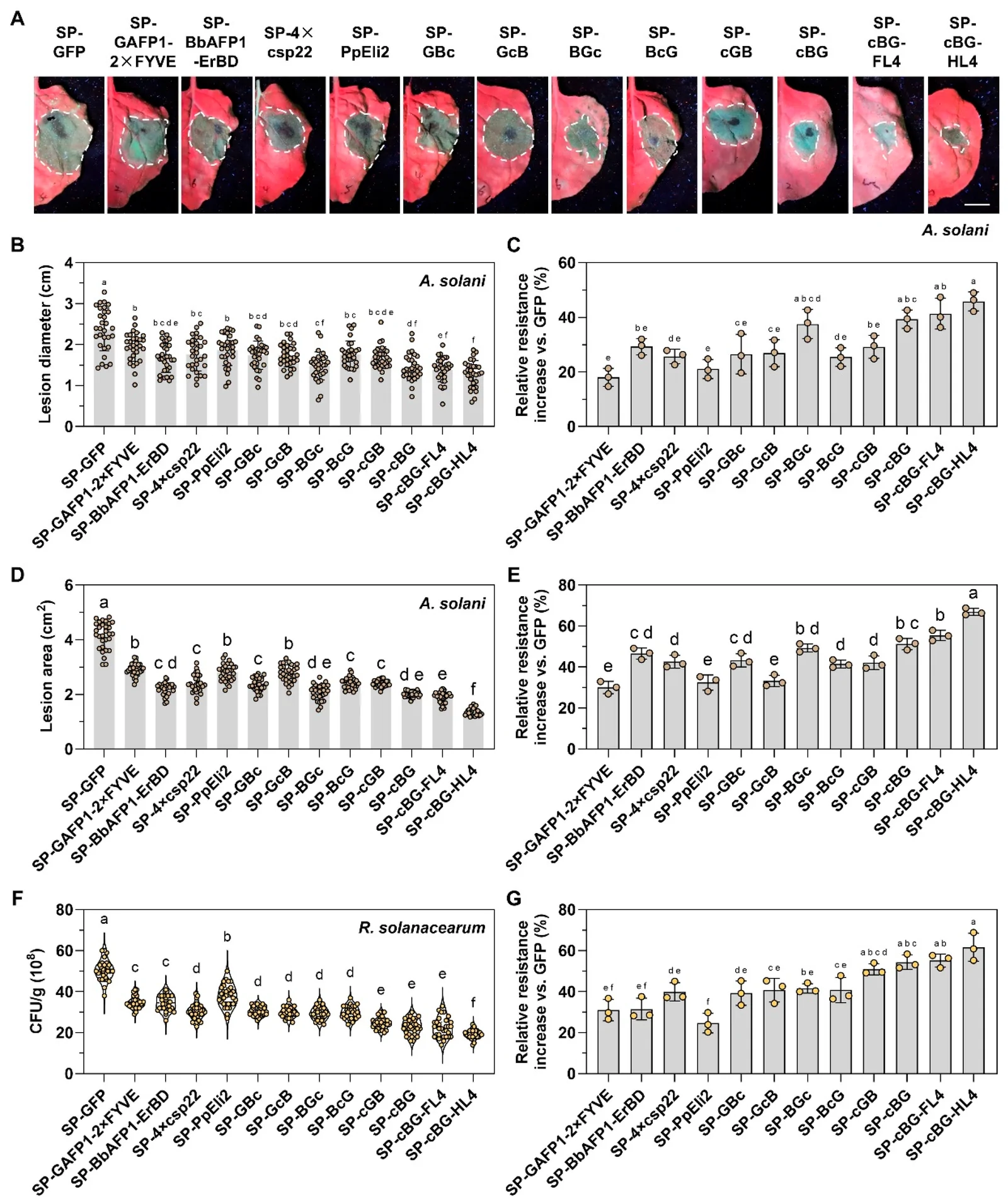
Evaluation of resistance conferred by 12 fusion constructs against *A. solani* and *R. solanacearum*: (**A**) Representative disease phenotypes of *N. benthamiana* leaves expressing indicated SP-fusion constructs at 4 dpi with *A. solani*. Scale bar, 1 cm. (**B**) Quantification of *A. solani* lesion diameter upon expression of different fusion constructs (*n* = 30 technical replicates). (**C**) Relative resistance increase (%) of each construct relative to the SP-GFP control, calculated from lesion-diameter data in (**B**) (*N* = 3 biological replicates). (**D**) Lesion area measurement of *A. solani*-challenged leaves for 12 fusion constructs (*n* = 30 technical replicates). (**E**) Relative resistance increase (%) normalized to SP-GFP control derived from lesion area statistics in (**D**) (*N* = 3 biological replicates). (**F**) Bacterial population quantification of *R. solanacearum* in leaf tissues expressing different fusion constructs (*n* = 30 technical replicates). (**G**) Relative resistance increase (%) compared with SP-GFP control, calculated from bacterial CFU counts in (**F**) (*N* = 3 biological replicates). Data are presented as mean ± SD. The Shapiro–Wilk test (α = 0.05) was used to assess normality and log-normality of data distributions across groups. One-way ANOVA was performed for normally distributed datasets, followed by Tukey’s multiple-comparison test for all pairwise comparisons. Different lowercase letters indicate significant differences among treatments (*p* < 0.05). All experiments were repeated three times with consistent results.

We further evaluated the broad-spectrum resistance conferred by the 12 fusion constructs against the fungal pathogen *A. solani* and the bacterial pathogen *R. solanacearum*. Upon *A. solani* inoculation, representative leaf phenotypes at 4 dpi showed varying degrees of lesion development among constructs (Figure 3A). Quantitative measurements of lesion diameter (Figure 3B) revealed that SP-cBG, SP-cBG-FL4, and SP-cBG-HL4 exhibited the smallest lesion diameters, indicating the best disease resistance. The relative resistance increases calculated from lesion diameter data showed that SP-cBG-HL4 ranked the highest, although its difference from SP-cBG and SP-cBG-FL4 was not statistically significant (Figure 3C). For lesion area measurements, SP-cBG-HL4 conferred the greatest reduction (Figure 3D), and the corresponding relative resistance increase was also the highest, with statistically significant differences compared to SP-cBG and SP-cBG-FL4 (Figure 3E). For bacterial resistance, we quantified *R. solanacearum* population sizes in leaf tissues expressing different constructs. CFU counts indicated that SP-cBG-HL4 exhibited the lowest bacterial titers (Figure 3F). The corresponding relative resistance increase ranked the highest, although its difference from SP-cGB, SP-cBG, and SP-cBG-FL4 was not statistically significant (Figure 3G). Collectively, these results suggest that SP-cBG-HL4 confers relatively superior resistance against *A. solani* and *R. solanacearum*, as reflected by smaller lesion areas and lower bacterial CFU counts, respectively.

### 3.4. Expression Analysis of Defense-Related Marker Genes Induced by 12 Fusion Constructs

To further validate the immune-inducing effects of the 12 fusion constructs, we extended our analysis from macroscopic disease phenotypes to molecular plant immune signaling. Plant defense responses are orchestrated by multiple interconnected signaling pathways. Among them, PAMP-triggered immunity (PTI) serves as the first line of inducible defense, while the salicylic acid (SA) and jasmonic acid/ethylene (JA/ET) pathways not only mediate basal resistance but are also deeply involved in effector-triggered immunity (ETI) against diverse pathogens. To comprehensively assess the activation of these pathways, we selected 6 well-established marker genes, including *CYP71D20* and *PTI5* for PTI, *PR1* and *PR2* for SA, and *PR3* and *PR4* for JA/ET, as indicators of the corresponding immune responses [19,25].

**Figure 4:**
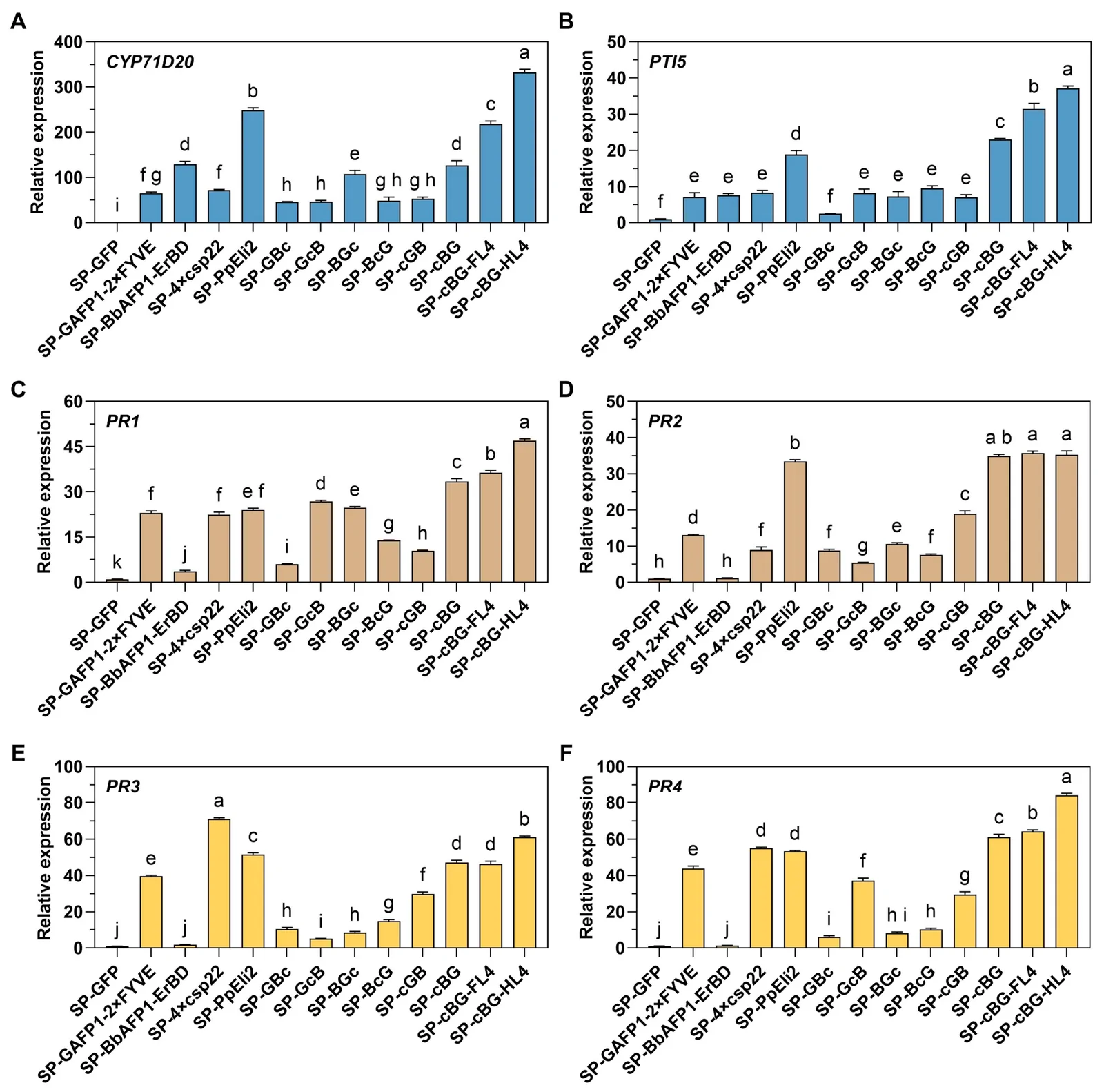
qRT-PCR analysis of defense-related marker gene transcript abundance in leaves expressing 12 fusion constructs: (**A**,**B**) Relative transcript levels of PTI-responsive marker genes *CYP71D20* and *PTI5*. (**C**,**D**) Relative expression of SA-pathway marker genes *PR1* and *PR2*. (**E**,**F**) Transcript accumulation of JA/ET-pathway marker genes *PR3* and *PR4*. Data are presented as mean ± SD, *N* = 3 independent biological replicates, and all experiments were repeated three times with consistent results. The Shapiro–Wilk test (α = 0.05) was applied to evaluate the normality and log-normality of data distributions across groups. One-way ANOVA was performed for normally distributed datasets, followed by Tukey’s multiple-comparison test for all pairwise comparisons. Different lowercase letters indicate significant differences among treatments (*p* < 0.05).

To assess the activation status of the PTI-, SA-, and JA-responsive marker genes, we examined the transcript levels of the corresponding marker genes in response to the different constructs. For the PTI pathway, the hallmark marker genes *CYP71D20* and *PTI5* were significantly upregulated by most constructs, with the highest expression levels observed in SP-cBG-HL4-expressing leaves (Figure 4A). For the SA-responsive gene *PR1*, SP-cBG-HL4 again showed the most pronounced accumulation (Figure 4C); however, for *PR2*, the highest expression levels were observed in SP-cBG, SP-cBG-FL4, and SP-cBG-HL4, with no significant differences among these three groups (Figure 4D). In the JA/ET pathway, for the reporter gene *PR3*, SP-4×csp22 exhibited the highest expression level, followed by SP-cBG-HL4 as the second highest (Figure 4E), whereas for *PR4*, it was consistently upregulated, with SP-cBG-HL4 ranking among the top groups (Figure 4F). Collectively, these gene expression analyses demonstrate that SP-cBG-HL4 triggers relatively optimal transcriptional upregulation of PTI marker genes among all tested constructs.

### 3.5. Integrated Summary of Resistance Induction and Phenotypic Performance of 12 Fusion Constructs

**Figure 5:**
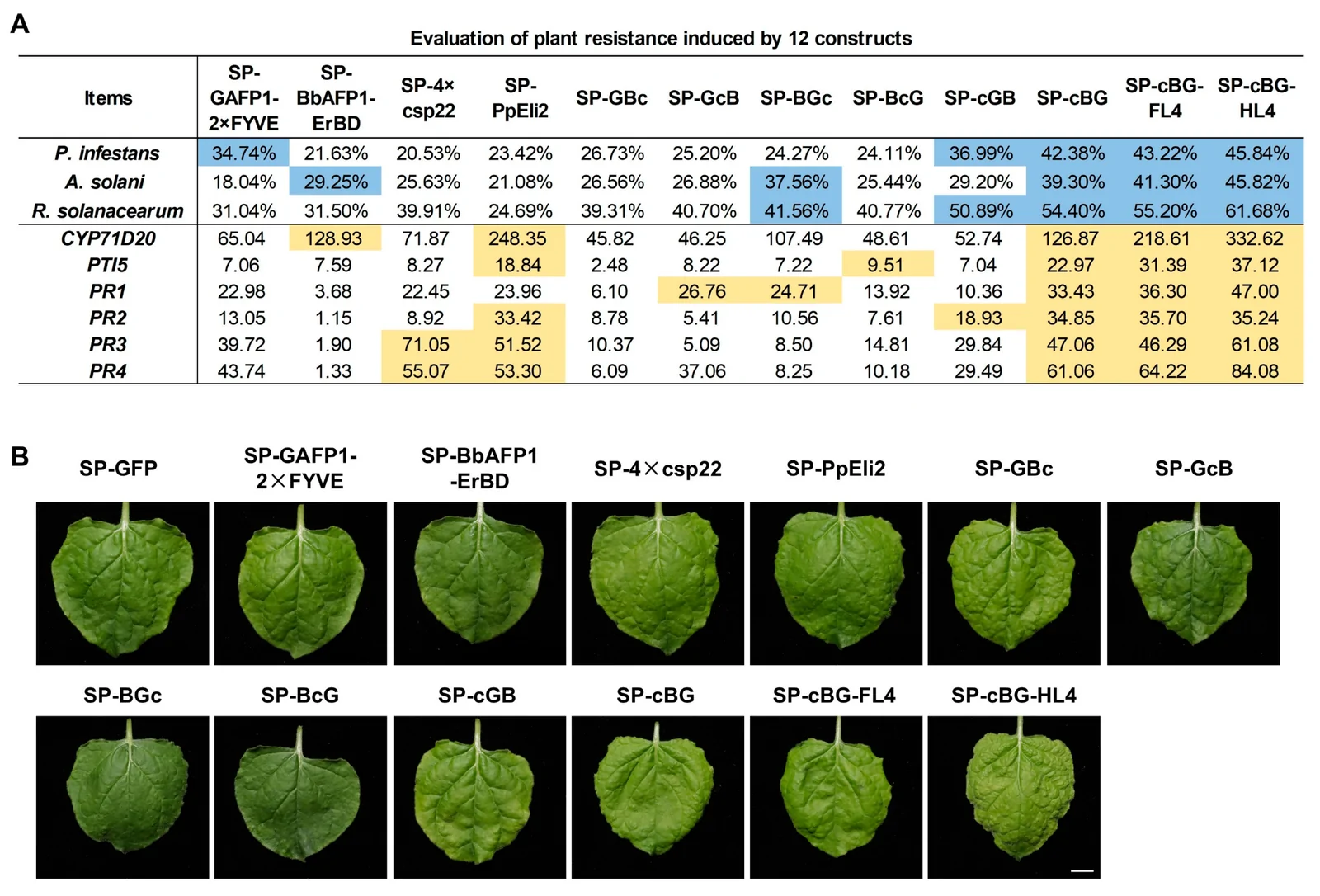
Summary of resistance-induction capacity and leaf phenotypic performance upon transient expression of 12 fusion constructs in *N. benthamiana*: (**A**) Statistical table summarizing relative pathogen-resistance induction rates and defense-gene expression fold-changes for 12 fusion constructs. Blue background marks the top five values of resistance induction efficiency, and yellow background highlights the top five highest upregulation folds of defense marker genes. (**B**) Representative leaf phenotypes of *N. benthamiana* transiently expressing the 12 fusion constructs in the absence of pathogen inoculation. Scale bar, 1 cm.

To provide an integrated assessment of the 12 fusion constructs, we summarized their relative pathogen resistance induction rates and defense gene expression folds in a statistical table (Figure 5A). Based on disease resistance against the three major pathogens, SP-cBG-HL4 consistently ranked first against *P. infestans*, *A. solani*, and *R. solanacearum*, followed by SP-cBG-FL4 and SP-cBG. The top five constructs against *P. infestans* were SP-cBG-HL4, SP-cBG-FL4, SP-cBG, SP-cGB, and SP-GAFP1-2×FYVE. For *A. solani*, the top five included SP-cBG-HL4, SP-cBG-FL4, SP-cBG, SP-BGc, and SP-BbAFP1-ErBD. Similarly, the top five against *R. solanacearum* were SP-cBG-HL4, SP-cBG-FL4, SP-cBG, SP-cGB, and SP-BGc. Regarding defense gene activation, SP-cBG-HL4 also displayed the highest induction folds for *CYP71D20*, *PTI5*, *PR1*, and *PR4*, with SP-cBG-FL4 and SP-cBG consistently ranking among the top tiers. Specifically, the top five for *CYP71D20* were SP-cBG-HL4, SP-PpEli2, SP-cBG-FL4, SP-BbAFP1-ErBD, and SP-cBG. For *PTI5*, the ranking was SP-cBG-HL4, SP-cBG-FL4, SP-cBG, SP-PpEli2, and SP-BcG. The *PR1* top five consisted of SP-cBG-HL4, SP-cBG-FL4, SP-cBG, SP-GcB, and SP-BGc. For *PR2*, the top five were SP-cBG-FL4, SP-cBG-HL4, SP-cBG, SP-PpEli2, and SP-cGB. For *PR3*, the leading constructs were SP-4×csp22, SP-cBG-HL4, SP-PpEli2, SP-cBG, and SP-cBG-FL4. For *PR4*, the top five were SP-cBG-HL4, SP-cBG-FL4, SP-cBG, SP-4×csp22, and SP-PpEli2 (Figure 5A). In addition, we evaluated the effects of transiently expressing the 12 fusion constructs on *N. benthamiana* leaves. As shown in the representative leaf images (Figure 5B), expression of all constructs in the absence of pathogen challenge did not result in any obvious adverse leaf phenotypes, indicating that these fusion proteins are well tolerated by *N. benthamiana*. Collectively, SP-cBG-HL4 is the relatively optimal construct among the 12 designs, ranking first in seven out of nine evaluated parameters, and it exhibited balanced broad-spectrum resistance, effective defense gene activation, and no obvious adverse effects on leaf phenotypes.

## 4. Discussion

In this study, we systematically evaluated the impact of modular order and linker design on the broad-spectrum resistance conferred by multi-module fusion constructs. By assembling four functional modules—GAFP1-2×FYVE (oomycete-inhibiting), BbAFP1-ErBD (fungal-suppressing), 4×csp22 (bacterial-derived PAMP), and PpEli2 (broad-spectrum MAMP)—into various tandem arrangements, with 4×csp22 and PpEli2 linked together owing to their common identity as extracellular effectors. We identified SP-cBG, SP-cBG-FL4, and SP-cBG-HL4 as the top-performing constructs against *P. infestans*, *A. solani*, and *R. solanacearum*, with SP-cBG-HL4 exhibiting the most balanced and comprehensive efficacy. Notably, the order of modules exerted a marked influence on disease resistance, as exemplified by the contrasting performance of SP-cBG and SP-BGc despite sharing identical components. This positional effect may be attributed to differences in translational efficiency, protein-folding stability, signal-peptide cleavage kinetics, or linker accessibility among module junctions. The observation that the incorporation of flexible (FL4) or rigid (HL4) linkers further enhanced the performance of SP-cBG-HL4 suggests that linker optimization may have played a role in enhancing the performance of this multi-domain protein. These findings are consistent with previous reports demonstrating that rational design of fusion proteins can potentiate the potential synergistic antimicrobial activities [23].

The transcriptional profiling of defense marker genes revealed that SP-cBG-HL4 induced the most comprehensive upregulation of marker genes associated with the PTI, SA, and JA/ET signaling pathways among all tested constructs. Specifically, it induced the highest expression levels of the PTI hallmark genes *CYP71D20* and *PTI5*, as well as the SA-responsive gene *PR1*, while also ranking among the top constructs for the JA/ET marker gene *PR4*. The strong immune activation observed in constructs containing PpEli2 is consistent with its known function as a MAMP recognized by the cell-surface receptor REli, which forms a complex with the co-receptor BAK1 to initiate downstream defense responses [18,26]. Interestingly, the single-module construct SP-4×csp22, which contains a bacterial PAMP derived from *R. solanacearum*, exhibited the highest upregulation of *PR3*, but did not rank among the top performers in terms of overall disease resistance across all three pathogens. In contrast, multi-module fusions that induced higher expression of marker genes across multiple signaling cascades—likely through potential synergistic crosstalk between PTI and SA/JA/ET pathways—conferred superior broad-spectrum resistance. This observation aligns with the prevailing view that coordinated upregulation of multiple defense-related genes contributes to more effective plant immunity [27,28].

Despite the promising results, several limitations should be acknowledged. First, all experiments were performed exclusively in *N. benthamiana*, and whether the observed effects can be extended to crop species—particularly potato, which is a major host of all three pathogens—remains to be determined [13,29]. Nevertheless, these findings provide a valuable proof of concept for future translational studies. Second, only a single isolate or race of each pathogen was tested, and the efficacy against other virulent strains or field populations requires further validation. Despite these constraints, our systematic comparison offers important insights into the engineering of multi-module fusion proteins for enhanced disease resistance.

## 5. Conclusions

In summary, this study systematically evaluated a suite of 12 signal peptide (SP)-fused recombinant constructs designed to confer broad-spectrum disease resistance against the oomycete *P. infestans*, the fungus *A. solani*, and the bacterium *R. solanacearum*—the three major pathogens threatening global potato production. Through modular rearrangement of four functionally distinct antimicrobial domains (GAFP1-2×FYVE, BbAFP1-ErBD, 4×csp22, and PpEli2), we identified the SP-cBG arrangement as the optimal multi-domain combination. More importantly, the subsequent introduction of a rigid linker (HL4) into this architecture (SP-cBG-HL4) further potentiated its resistance efficacy against all three pathogen classes, with the strongest suppression of disease lesions and the strongest overall defense-associated transcriptional response. Together, these findings demonstrate that strategic module ordering and linker-mediated modular combinatorial optimization represent a powerful and rational engineering approach to create multi-functional antimicrobial proteins. This strategy effectively addresses the long-standing challenge of simultaneously managing oomycete, fungal, and bacterial diseases; nevertheless, its translational potential in potato is a promising avenue that requires direct testing in this crop.

## Figures and Tables

**Table 1:** List of primers used in this study.

Primer Name	Primer 5′-3′
qNbCyp71D20-F	GTTGACGCCATTGTTGAG
qNbCyp71D20-R	ATCTTCGCCTCCTAATGC
qNbPti5-F	CCTCCAAGTTTGAGCTCGGATAGT
qNbPti5-R	CCAAGAAATTCTCCATGCACTCTGTC
qNbPR1-F	GTGGACACTATACTCAGGTG
qNbPR1-R	TCCAACTTGGAATCAAAGGG
qNbPR2-F	AGGTGTTTGCTATGGAATGC
qNbPR2-R	TCTGTACCCACCATCTTGC
qNbPR3-F	CAATGCCTTTATCAATGCTG
qNbPR3-R	AGTAGTCACCTGGGCTACCT
qNbPR4-F	GATGCTTGAGGGTGACGA
qNbPR4-R	ATAGCCCACTCCATTTGT
qNbEf1a-F	AGAGGCCCTCAGACAAAC
qNbEf1a-R	TAGGTCCAAAGGTCACAA

## Data Availability

The original contributions presented in this study are included in the article. Further inquiries can be directed to the corresponding authors.
